# Leakages in District Heating Networks—Model-Based Data Set Quality Assessment and Localization

**DOI:** 10.3390/s22145300

**Published:** 2022-07-15

**Authors:** Kai Vahldiek, Bernd Rüger, Frank Klawonn

**Affiliations:** 1 Institute for Information Engineering, Ostfalia University of Applied Sciences, Salzdahlumer Str. 46/48, 38302 Wolfenbuttel, Germany; f.klawonn@ostfalia.de; 2 SWM Services GmbH, Emmy-Noether-Str. 2, 80992 Munchen, Germany; rueger.bernd@swm.de

**Keywords:** district heating network, leakage localization, sensors, data analysis, quality assessment, measurement data

## Abstract

Large spontaneous leakages in district heating networks (DHNs) require a separation of the affected network part, as interruption of the heat supply is imminent. Measurement data of 22 real events was analyzed for localization, but suitable results were not always achieved. In this paper, the reasons are investigated and a model for data evaluation (MoFoDatEv) is developed for further insights. This contains prior knowledge and a simplified physical model for the reaction of the DHN in the case of a large spontaneous leakage. A model like this does not exist so far. It determines the time point and the duration of the pressure drop of the pressure wave which is caused by such leakages. Both parameters and the evaluation time frame are optimized for each event separately. The quality assessment leads to a categorization of the events based on several parameters, and correlations between the pressure and the refill mass flow are found. A minimum leakage size is deduced for successful evaluation. Furthermore, MoFoDatEv can also be used for leakage localization directly, combining two steps from previous publications. Therefore, more data contribute to the result. The application is conducted with artificial data to prove the model concept, and also with real measurement data.

## 1. Introduction

**Figure 1:**
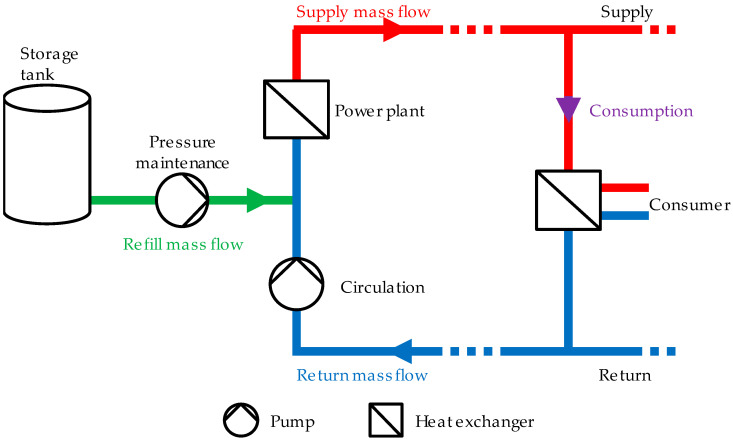
Schematic representation of a district heating network (Reprinted with permission from Ref. [3]. 2022, Vahldiek).

The energy transition can only succeed if the change to renewable energies is also pursued for the supply of heat. District heating networks (DHNs) have a potentially important role for the green transition [1]. During the whole year, DHNs are used to supply customers with energy. An uninterrupted energy supply is required. However, there is a risk of leakages because the hot fluid medium is transported under high levels of pressure [2]. In Figure 1, a schematic representation of a DHN is shown.

DHNs, as a special form of thermo-hydraulic network, are used to supply customers with energy. The whole system is closed, pressurized, and operated with deionized water (a special, treated form of water) as a medium. Supply pipes and mass flow are drawn in red and return in blue. With a heat exchanger, the consumers obtain the energy from the network. The pressure difference between supply and return drives the mass flow through the heat exchanger, and the supply temperature is reduced to the return temperature. To this end, the network operator has to ensure a minimal pressure difference and supply temperature, and the customer has to cool down the medium to a maximal allowed return temperature. The pressure difference is regulated by the circulation pump. If it is too low, the circulation pump power is increased, and if it is too high, pump power is decreased. The pressure maintenance pump ensures a certain pressure level throughout the whole network. At the supply site, the cooled-down medium is heated up again by a heat exchanger. Typical temperatures for the supply and the return are 120 °C and 70 °C, respectively. This leads to a temperature difference of 50 °C. Typical pressures at the power plant are around 18 bar in the supply and around 5 bar in the return. Several heat sources are possible. A power plant is depicted in Figure 1. When transporting the medium from the power plant to the consumer, no medium should be lost in order to ensure an uninterrupted supply. However, leakages can occur within the pipes. In the case of a large, spontaneous leakage, loss of the transport medium can be compensated only for a certain period of time. The replenishment is performed via the refill mass flow (green), but the available quantity for this is limited. The refill is necessary to maintain the prevailing pressure in the entire network and to keep the medium liquid due to the high supply temperature [3]. However, if the loss of medium is too large, it may be necessary to stop network operation. Then, a fast localization of the leakage is an important task to enable further network operation. Network shutdown can be prevented by taking appropriate countermeasures, such as disconnecting the damaged network section using exclusion areas (EA). To be able to use these measures in a targeted manner, the leakage must be assigned to one of the EAs as quickly and accurately as possible.

Generally, a distinction is made between small creeping and large spontaneous leakages. All small leakages which are present will be replenished continuously as long as the summed-up refill mass flow is not too large. They do not have any impact on the consumer and the heat generation of the supplier. Nevertheless, heat and water losses are present. Small leakages are hard to detect and localize as their impact on measurement values is very small. On the other hand, there are large, spontaneous leakages, which can only be replenished for a very short time of about 15 min to 3 h. During this time, leakage localization and separation of the affected network part has to be performed. To this end, it is beneficial that these leakages are clearly reflected in the measurement data and a pressure wave is likely to be present. Only large spontaneous leakages are in the scope of this paper. If it is not possible to refill the medium anymore, the pressure in the system drops to zero and the hot medium evaporates. Thus, circulation stops. The pipes in the heat generator of the supplier cannot be cooled anymore and will be destroyed. The consumers cannot be further supplied with heat. At the damaged spot, the exiting medium endangers people and environment.

Previous publications picked up the evaluation chain from detection to separation with a focus on a framework employing test leakages (TL) and a performance criterion (PC) as a measure when evaluating the negative pressure wave caused by the leakage. Attribution of one large spontaneous leakage to EAs works reasonably well if the pressure drop time points (PDTP) can be determined precisely enough. The minimum number and optimal placement of sensors was examined. Subsequently, different algorithms for extraction of the PDTPs were assessed employing 22 real data sets. The overall best time frames $\left[T_{1},T_{2}\right]$ for practical application for each algorithm were determined. During this assessment, the findings showed, data sets can be evaluated with different PC values ranging from 100.0% (best) to 3.6% (worst), where 100% means the EA affected by the leakage was ranked first and 3.6% means it was ranked last. A proposal was given to look more deeply into data set quality to gain an understanding of what leads to this difference in the quality of the results when evaluating the pressure waves within the different data sets.

The aim of this paper is the development and application of a model for data evaluation (MoFoDatEv) to evaluate data sets of several real events. Prior knowledge is used for the development of the model. MoFoDatEv employs a simplified physical model for the reaction of the DHN in the case of a large spontaneous leakage. It is assumed that the pressure drop occurring at the damaged spot propagates through the network without attenuation due to network size. The pressure curve is modelled as a piecewise-defined function with constant start and end values and a linear decrease in between at all sensors. Pressure wave travel times due to the position of leakage and sensors are taken into account. The model will be applied to each event and the values of the time point for the start of the pressure drop $t_{1}$ and the duration of the pressure drop $\Delta_{p,t}$ will be determined. After a rough determination of these values, they will also be optimized to find the best suitable values. The best suitable values are found if the model fits the real data with the smallest possible deviation. After the evaluation, the model is also used to localize leakages directly within the network. In prior publications, there were two steps necessary: the determination of the PDTP [3] and the attribution to EA to separate the damaged network part [4]. With MoFoDatEv, both steps will be combined as a novelty and first results for artificial and real data will be shown.

The paper is divided into six sections. After the introduction, Section 2 presents related work. In Section 3, the background and motivation are given. MoFoDatEv is described in detail in Section 4. Then, the results of the data evaluation are presented as well as a new method for leakage localization in Section 5, before Section 6 briefly concludes.

## 2. Related Work

For the evaluation of pressure data within a DHN, no literature is available to our knowledge. Nevertheless, we provide a brief overview on some literature that could be relevant within other contexts and we apply some ideas from them to our problem. Some literature about the data quality assessment of various sensors is available, which is presented briefly to classify our model. About leakage localization within a DHN, there is varied literature available, which will be presented. Based on these ideas, our method will use an improved approach for the leakage localization.

In Iantovics’ paper [5], a method for data quality assessment of synthetic industrial data is presented. Synthetic data are necessary because sometimes it is difficult to obtain real data from sensors or machines and use them in another context. However, the simulated data are limited in usage, based on their poor quality. An approach is proposed for a mathematically grounded data quality assessment to analyze the predictive power of the variables. Assumptions are made which have to be passed by the synthetic data. Furthermore, various indicators for assessing the quality such as e.g., sensitivity, specificity, and accuracy are introduced.

In Smith’s paper [6], the sensor data quality is assessed by an automated online Bayesian framework. This is necessary because the assessment of the determination of a sensor’s quality is critical for usage in real-time applications. A framework is proposed to represent the causes and the quality state of individual sensor errors. Using this approach, a probabilistic quality assessment is established to show the uncertainty of sequentially correlated sensor readings.

In Timm’s paper [7], an automated data quality assessment of marine sensors is presented. The automated data collection led to an increase in the amount of available data. Due to the demand of real-time applications, an automated quality assessment of the data is necessary. This ensures that the data are fit for purpose. A method is proposed to evaluate the data on a quality scale and not, as is often the case, only to make a binary classification between good and bad data. The method is tested on data from marine sensors in a real-time platform.

In Garcia’s paper [8], quality sensors are used to ensure compliance with quality standards in a water distribution system. The sensors have to be maintained frequently in order to work correctly and reliably. During maintenance, the health status of the sensors should also be taken into account. In addition, the collected data should be validated before starting the prognosis of the sensors. Therefore, a method is presented that uses data analysis to detect water quality sensor failures and water quality events. By means of a prognosis system, it is also possible to evaluate the sensors. For this purpose, the time series of the collected data by the quality sensors are analyzed within the system.

In Rahman’s paper [9], the quality assessment of sensor data is performed by a novel machine learning approach. The quality of sensor data is indicated by discrete flags to indicate the degree of associated uncertainty. However, the degree of uncertainty varies because the flags are mainly used by experts. Due to the increasing amount of sensor data, manual assignment is not possible without error. Data with poor quality will lead to an imbalance in data classification. Therefore, a cluster-oriented sampling approach is presented to solve the imbalance. It can also be used to train multiple classifiers to improve the overall classification accuracy. Marin sensor data are used to test the ensemble classification framework. The results show that the framework agrees with the expert estimation with high accuracy.

In Vahldiek’s paper [3], the data of several real events in cases of leakage within a DHN are evaluated. For each event, different algorithms are applied to detect the PDTP. The optimal algorithms are found. Furthermore, the overall best time frames, where the algorithms deliver the best results, are also investigated. With the correct detection of the PDTP, the attribution to the EA can be performed to localize the leakage. For further real events, an optimal algorithm, with its overall best time frame to evaluate the pressure data, is suggested.

In Vahldiek’s paper [4], a framework is developed and presented to perform the attribution to EA: This attribution is calculated based on the existing PDTPs extracted from measurement data. For assessment of the quality of the leakage localization, a performance criterion is introduced. The framework can be used for optimal sensor placement and also for the investigation of noise. For up to five sensors, an optimal sensor placement is presented. In addition, the influence of the noise of the pressure drop time points is evaluated. For different numbers of optimally placed sensors, acceptable noise levels are investigated to allow sufficient leakage localization quality. The results are presented and discussed.

In Rüger’s paper [10], an online prototype was developed and presented to localize leakages in a DHN by evaluating the resulting pressure wave. The localization is performed in two steps. First, the PDTP is estimated for each sensor. With the time information of the detection the pressure wave at each sensor, the attribution to the EA is applied. For the detection of the PDTP, an implemented algorithm is used. The affected EA can be separated from the rest of the network after the attribution to EA. The prototype can localize leakages and, for a first real event, plausible results are achieved.

In Vahldiek’s paper [11], real measurement data are evaluated with three different model- and data-driven approaches. The first approach evaluates the pressure wave in the case of a leakage which propagates throughout the entire network. The second and third approaches are purely data driven. Machine learning models are used within the second approach to localize leakages. The third approach uses available measurement data for numerical computation to determine the network state and the leakage localization. Two real events are evaluated to demonstrate the applicability of the approaches. All three methods are well suited for leakage localization, but the approaches have specific advantages and disadvantages for each event.

In Pierl’s paper [12], three different model- and data-driven approaches evaluate artificial measurement data in a DHN for leakage localization. Two methods can only be used within a new steady state after leakage occurrence whereas the third approach evaluates the resulting pressure wave. The first two methods, a model-based numeric–analytical and a machine learning model, use multiple measurement data like pressure, flow rate, and temperature. The third method is applied on pressure data for detection of the pressure wave. Each approach is presented and applied to the simulated measurement data. Furthermore, for each approach, the influence of random measurement noise is investigated and plausible results are achieved.

In Yuan’s paper [13], an analysis and evaluation of the operation data in a district heating substation for a consumption prediction model is performed. For building the prediction model, the steps of data cleaning, model establishment, and effect evaluation are necessary. Therefore, the problem of missing values is addressed and a solution is suggested. The model used for evaluating the data is based on the calculation of the error between the actual and predicted value. If the actual value is not on the same level as the on-demand value, the method cannot accurately evaluate. This leads to a limited applicability of the prediction model to the existing data.

In Zhou’s paper [14], a comparison of different methods for leakage localization in a DHN is performed. A distinction is made between data-driven, physical-model-based, and unmanned airborne infrared thermography methods. The advantages and disadvantages of the application are discussed. If a sufficient database is available, the data-driven methods can easily be applied to other networks.

In Valinčius’ paper [15], a method for leakage localization in a DHN is presented. The basis is the pressure data from the different sensors within the network. In the case of a leakage, the negative pressure wave is evaluated to localize the correct location. In addition, the optimal data record time for the sensors is presented.

In addition, other, different approaches for leakage localization in DHNs are available. A data-driven method is presented in Xue’s paper [16]. There is the training of a decision-tree-based machine learning algorithm which uses generated data based on a physical model. The collected data from pressure and flow sensors within the investigated DHN are used to detect the leakage. However, this method depends on the availability of pressure sensors in the network. In contrast, different approaches for pipelines are developed and compared in Zaman’s paper [17]. Model-based and data-driven methods are used within an algorithm to detect leakages. In Liu’s paper [18], a physical-model-based algorithm for the detection is utilized.

The literature review has shown that there are some useful components in every presented publication, which are also taken up in this paper. From [5,6,7], the idea of an indicator to classify good and bad data is used. In this paper, a quality measure will be introduced to compare the different data sets. The collected data from [8] are used to identify sensor failures and water quality events. This is comparable to the application of this paper, where leakages are the events which have to be identified by evaluating the sensor data. The accuracy is based on the quality of the data, which is why an assessment is necessary. The machine learning approach from [9] classified the different data sets, and this is also included in our proposed method. In [13], the evaluation of the data is also applied as in this paper. Furthermore, the model used calculated the error between the actual and predicted value of the heat consumption. For our model, there will be also an error calculated between the measurement data and the fitted data. In [14,15], useful methods for leakage localization are applied likewise as in [16] but with a different aspect of the application of a machine learning algorithm. Ideas from [17,18] for leakage detection in pipelines are considered here but pipelines are not directly comparable with a DHN because a DHN is a closed system. The negative pressure wave is evaluated, which is also the basis for MoFoDatEv for the leakage localization. In [10,11,12], the presented evaluation of the pressure wave is divided into two steps: The determination of the PDTPs and the attribution to the EAs. The best suitable algorithm for the PDTP detection is presented in [3] and the required framework in [4]. In this paper, we borrow from these ideas, but an approach is proposed to combine these two steps.

## 3. Basics and Motivation

**Figure 2:**
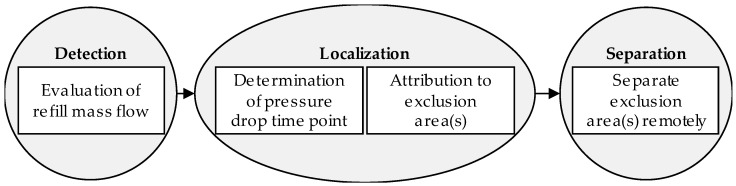
Emergency management steps in the case of a large spontaneous leakage with imminent shutdown of the network (Reprinted with permission from Ref. [3]. 2022, Vahldiek).

In the case of a large, spontaneous leakage where a network shutdown is imminent, Figure 2 shows three very generic but still relevant steps with detection, localization, and separation. To secure safe network operation, the damaged network part has to be separated as fast as possible [3]. Two of these steps are already established in practice. The first step, leakage detection, has been realized reliably by monitoring the refill mass flow in real time, as described in [10,19]. A fast separation of the damaged network part is possible due to the installed EAs. However, the step of localization of the leakage is the crucial one to separate the right EA from the rest of the network.

In the case of a large spontaneous leakage where a network shutdown is imminent, Figure 2 shows the three main steps. The detection of the leakage can be realized reliably by monitoring the refill mass flow in real time like described in [10,19]. Then the leakage detection time point (LDTP) can be estimated. Based on the installed EAs, a fast separation of the damaged network part is possible. The localization is divided into two more sub-steps.

Localizing the leakage position in the network is a demanding task. The localization is performed by evaluating the negative pressure wave. In the case of a leakage, the pressure drops at the leakage position and a negative pressure wave propagates through the entire network and reaches every sensor at a different time point. In the first sub-step for localization, the PDTPs for each sensor are estimated with an algorithm, when the pressure wave is registered by the sensor as described in [3]. In the second step, these PDTPs are used to attribute the leakage location to the EAs [4]. Both steps are already implemented in a prototype application in [10] which runs 24/7 without human interference. A challenge is the improvement of localization results.

Several real events are evaluated in [3]. Different algorithms to detect the PDTPs are investigated and applied to these events. Furthermore, different time frames for the application of the algorithms are examined. Based on a PC, the different algorithms are compared. Within each optimal time frame for the combination of event and algorithm, the leakage will be localized correctly in most of the cases. However, even for one algorithm, the optimal time frame differs depending on the events. For practical application, it is not admissible to use different time frames. A single time frame has to be chosen for each algorithm prior to evaluation of new data sets. Based on the individual time frames of each algorithm, the overall best time frame is selected. Within this overall best time frame, several events were evaluated again with different algorithms. The Bayesian single change point (BCP) algorithm delivered the best localization results and is implemented in the aforementioned prototype in the next step. The division of the pressure curve into multiple blocks and the use of Bayesian methods to estimate the change point as the required PDTP is an advantage of this algorithm [20]. Larger differences within the pressure curves can be better determined with these blocks. Therefore, the PDTPs are very well determinable and the attribution to EA also performs very well. However, the rating of the algorithms might change if further data becomes available or insight into data quality issues is gained. Further, it was recommended to look deeply into data set quality in [3].

**Figure 3:**
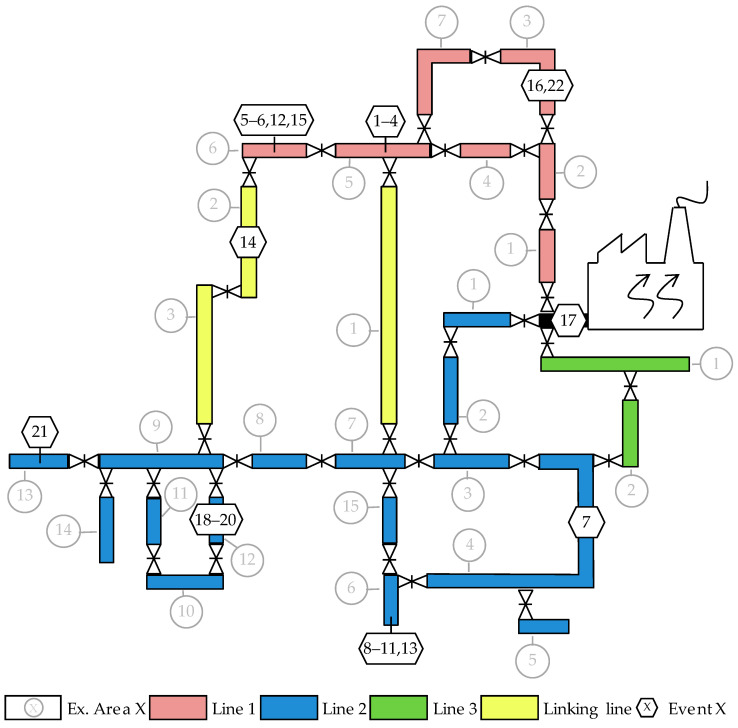
Representation of locations in the investigated network for each event.

In this paper, data set quality of the events is a main scope. To this end, an idealized model for the pressure curves is created and employed. In general, pressure curves belonging to one event are similar and shifted temporarily at every sensor but unique in shape for each event. Nevertheless, a generalized model should be determined. This is because the pressure wave is registered by the respective sensor at a different time point depending on the location of the leakage. As described in [21], the shape of the pressure wave is influenced by superpositions of the reflected portions of the pressure wave and the portions travelling along different ways in a complex network. This constitutes an additional challenge to finding a generalized model. The main idea is to find the right generalized model for the trend of the pressure data in the case of a leakage, which is a demanding task. Additionally, which evaluation time frame should be selected to evaluate the pressure data is also an important aspect. For practical application, a concrete time frame has to be chosen prior to data evaluation. Within this time frame, a possible generalized model can be applied to localize the leakage. However, before or after the LDTP, there could also be other processes in the DHN which have an influence on the pressure curves. Altogether, data evaluation and therefore leakage localization based on the pressure curves is a challenging task. Several real events were already evaluated. Measurement data of the necessary quality to be able to perform evaluations such as in this paper has been recorded since 2017. The last known large spontaneous leakages occurred in 2003 and 2010. Hence, data for refill processes is used because those events are assumed to act like a large spontaneous leakage on the entire network. The normal pressure difference between medium and environment is 5–18 bar. In the case of a leakage, the pressure difference is very large, so that a fast and drastic damage process is most likely. Hence, the temporal course of the mass flow through the damaged spot or through the valve in the case of a refill process is assumed to be equal. This assumption is supported by a smaller real leakage with properties which match very well to the events considered here ($\Delta_{m}$ = 34.1 $m^{3}/h$, $g_{m}$ = 81.2 $m^{3}/h/\min$; this data set was not examined as the pressure wave evaluation failed). In this case, a compensation pipe burst and the leakage rate was limited due to a casing pipe. In this paper, 22 real events are considered and the locations in the network are presented in Figure 3. For now, no further events are available.

The figure shows that most of the real events are in the EAs line 2 No. 6, with five events. In addition, EA line 1 No. 5 and No. 6 contains four events each. Sometimes, it is the same refill process at one location which is divided into several events (e.g., events 1–4). This is due to the valve opening, which is performed stepwise with waiting times in between. Each step causes a pressure wave which can be evaluated. The other events are distributed around the network. In Table 1, the 22 real events from Figure 1 are presented in detail, and they will be evaluated in the following.

With this current paper, more insights about the data quality should be given for the unique events. The PC value is different for each event when the BCP algorithm from [20] and used in [3] is applied within its overall best time frame $\left[T_{1},\;T_{2}\right]=\left[-100,\;30\right]$. A high PC value indicates that the affected EA is one of the first places within the ranking. A lower PC value shows a worse ranking. For the events 5, 9, 11, and 13, the affected EA is ranked in the first place. Furthermore, the BCP algorithm ranked the affected EA for nine events (5, 9, 11, 12, 13, 14, 19, 20, 22) in the first three places. However, some events have a worse PC of only 35.7%, which indicates a worse place in the ranking. Further investigation should be focused on the different results and the reasons for that. One reason could be the data quality of the different events. Therefore, the data are evaluated in more detail. The peculiarity of the pressure wave and the change in pressure around the LDTP is investigated. The degree and gradient of pressure level difference are determined. All of this is addressed with the generalized model.

## 4. Model for Data Evaluation (MoFoDatEv)

The basis of the developed model for quality assessment and leakage localization uses a model concept involving the physical process in case of a leakage. The developed model is called MoFoDatEv and is implemented in the open source software R [22]. The following two subsections define the basics and the development of MoFoDatEv to assess the quality of real events and also for leakage localization.

### 4.1. Definition of Quality Measures

For the data evaluation of several real events, MoFoDatEv uses the pressure data of each sensor. The model is based on assumptions:

1. Before and after the pressure drop, the measured pressures are (approximately) constant.
2. The pressure wave reaches each sensor with a temporal shift depending on the sensor’s position.
3. The level of the pressure drop and its gradient are (approximately) constant.

The assumptions lead to a simplified model. Pressure wave attenuation and superpositions of the reflected pressure wave portions are neglected. The leakage leads to a local pressure drop that spreads through the whole network. Based on its position in the network, the pressure wave reaches each sensor at a different time point. Because the network has a small spatial extension, the attenuation should be zero. As mentioned before, the pressure drops in the case of a leakage. Therefore, the pressure value drops from a higher start value to a lower end value. Thus, negative gradients are prohibited and end values higher than the start values are not allowed. MoFoDatEv is developed to check the fitting of the generalized model to the measurement data of each event. Additionally, three quality criterions were introduced to quantify the result of the model with the measurement data.

- Quality measure (QM) to assess the evaluability.
- Time range to apply the model.
- Temporal correlation between the PDTP and the refill mass flow.

**Figure 4:**
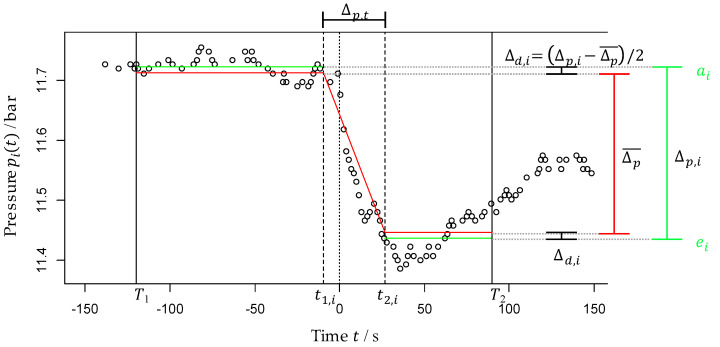
Example for sensor i with MoFoDatEv for data evaluation.

The first quality criterion indicates that, if QM is equal to zero, then the measurement data and the model match perfectly. The larger QM becomes, the worse the data set and the model match to each other. The second criterion is the usage of the time range in which the data set corresponds to the model. Ideally, this applies to the entire evaluation time frame of 300 s around the LDTP. The third criterion is the temporal correlation between the PDTP and the refill mass flow. Due to the network size and the speed of sound, there should be only a small difference. Figure 4 gives an overview of the application of MoFoDatEv and all relevant parameters.

The figure shows the application of MoFoDatEv to the sensor i of the data set from one event. Some prior knowledge and assumptions are used for the model. It is assumed that the pressure drop can be modelled as a piecewise-defined function with constant start and end values and a linear decrease in the pressure in between. The fit function of the model needs the LDTP to be at time point 0 and the data are presented within 300 s around this LDTP. In Figure 4, the evaluation time frame is defined by $T_{1}$ and $T_{2}$, with $T_{1}=-120$ and $T_{2}=90$. Within this time frame, the fit function (red lines) is determined. The pressure drops in between $t_{1,i}$ and $t_{2,i}$. The first model assumption is used in Formulas (1) and (2), where the start values $a_{i}$ and end values $e_{i}$ are the mean values of all data points $p_{i}\left(t\right)$ within the time frame $\left[T_{1},t_{1,i}\right]$ and $\left[t_{2,i},T_{2}\right]$, respectively (green lines).

(1) $$a_{i}=\bar{p_{i}\left(t\right)}\;\mathrm{in}\;[T_{1},\;t_{1,i}]$$

(2) $$e_{i}=\bar{p_{i}\left(t\right)}\;\mathrm{in}\;[t_{2,i},\;T_{2}]$$

For the second model assumption, it is defined that the pressure wave originating from the leakage reaches each sensor with a time delay of $\Delta_{i,L}$. The sensor located next to the leakage position has a time difference of $\Delta_{i,L}=0$ by definition. All other sensors are normalized to that sensor. Therefore, the values for $t_{1,i}$ and $t_{2,i}$ are calculated with Formulas (3) and (4). The time difference between these two points is defined as $\Delta_{p,t}$.

(3) $$t_{1,i}=t_{1}+\Delta_{i,L}$$

(4) $$t_{2,i}=t_{1,i}+\Delta_{p,t}=t_{1}+\Delta_{i,L}+\Delta_{p,t}$$

In Figure 4, the values are $t_{1}=-10.27$ and $t_{2}=36.06$. The last model assumption indicates that the level of the pressure drop is constant for all sensors within an event. The pressure level difference of each sensor is defined by $\Delta_{p,i}$, with the difference of $a_{i}$ and $e_{i}$. Furthermore, the pressure level difference for the whole event and the number of sensors N is denoted by $\bar{\Delta_{p}}$ according to Formulas (5) and (6). For one event, only sensors which have at least 15 data points per 60 s in the evaluation time frame $\left[T_{1},T_{2}\right]$ are considered.

(5) $$\Delta_{p,i}=a_{i}-e_{i}$$

(6) $$\bar{\Delta_{p}}=\sum_{i=1}^{N}\frac{\Delta_{p,i}}{N}$$

As $\bar{\Delta_{p}}$ is set to be equal to all sensors i, the fitted curve does not match $a_{i}$ and $e_{i}$. The decision was made to distribute the difference $\Delta_{d,i}$ equally.

(7) $$\Delta_{d,i}=\left(\Delta_{p,i}-\bar{\Delta_{p}}\right)/2$$

With the pressure level difference $\bar{\Delta_{p}}$ and the duration of the pressure drop $\Delta_{p,t}$, the gradient $g_{p}$ can be estimated with Formula (8).

(8) $$g_{p}=\frac{\bar{\Delta_{p}}}{\Delta_{p,t}}$$

With the application of MoFoDatEv, the introduced QM is used to assess the measurement data and the fitted data with the model. For each data point of sensor i, the mean squared error between the measurement $p_{i}\left(t\right)$ (black circles) and the fitted $q_{i}\left(t\right)$ data points (red line) is calculated. Then, for each sensor i, having $dp_{i}$ data points, $Q_{L,i}$ is determined according to Formula (9). With this, the quality of the data of the sensor i corresponding to the model was evaluated.

(9) $$Q_{L,i}=\frac{\sum {\left(p_{i}\left(t\right)-q_{i}\left(t\right)\right)}^{2}}{dp_{i}}$$

For all sensors used for each event, the $Q_{L,i}$ were also weighted with the overall evaluated data points $dp_{all}$ for the evaluation to define the $Q_{L}$ in Formula (10). This leads to an indication of the general quality of the fit for the whole dataset and the model.

(10) $$Q_{L}=\frac{\sum Q_{L,i}*dp_{i}}{dp_{all}}$$

MoFoDatEv is applied to each event with different values for the values $T_{1}$, $t_{1}$, $t_{2}=t_{1}+\Delta_{p,t}$ and $T_{2}$. The values of $T_{1}$ are in the range of $T_{1}\in \left\{-120,-110,\ldots ,-20\right\}$, and for $T_{2}$, in $T_{2}\in \left\{20,30,\ldots ,120\right\}$. For each sensor, the start of the pressure drop is determined with the choice of $t_{1}$ in a grid of $t_{1}\in \left\{-\left|T_{1}\right|*0.95,\ldots ,10\right\}$ with steps of 10. For the time difference $\Delta_{p,t}$, the grid is defined by $\Delta_{p,t}\in \left\{0.25,\ldots ,\left(T_{2}*0.95-t_{1}-0.25\right)\right\}$. Within these ranges, parameters are searched for which lead to the best concordance between MoFoDatEv and the data set. In general, the larger the evaluation time frame defined by $T_{1}$ and $T_{2}$, the better the model assumptions are applicable. However, these parameters are not as important as the other two defined in MoFoDatEv. For a given evaluation time frame $\left[T1,\;T2\right]$, $t_{1}$ and $\Delta_{p,t}$ (and thus $t_{2}$) have to be determined to achieve an optimized minimal value for $QM_{all}$. This means that the start of the pressure drop $t_{1}$ and its duration $\Delta_{p,t}$ have to be extracted from the data set.

The first level of the procedure is to generate a rough map for each value combination of $T_{1}$ and $T_{2}$ with the corresponding combination of $t_{1}$ and $\Delta_{p,t}$. The second level is also the generation of a rough map for the parameters $t_{1}$ and $\Delta_{p,t}$. Out of these two start value searches, the best combination of $t_{1}$ and $\Delta_{p,t}$, with the smallest $Q_{L}$, are the starting values for the optimization. For the optimization, the algorithm of Nelder and Mead is used [23]. The algorithm uses a simplex method for function minimization, which is very robust. Then, for each combination of $T_{1}$ and $T_{2}$, optimized values for the parameters $t_{1}$ and $\Delta_{p,t}$ are available and the best combination is selected with the best fitting results to the measurement data. After this selection and the fitting of MoFoDatEv to the measurement data, not only $\left[T1,\;T2\right]$ and their optimized parameters $t_{1}$ and $\Delta_{p,t}$ are available for interpretation, but also further parameters such as $\bar{\Delta_{p}}$ are considered.

### 4.2. Application for Leakage Localization

After the basics and the development of MoFoDatEv for quality assessment, some more information is needed to localize leakages. The same model with the same model assumptions and quality criterions is used for leakage localization. Previous publications have shown that two steps are necessary for the localization [4,10,11,12,21]. The first step is the determination of the PDTP [3]. Within the second step, this information is used to identify the EA most likely to contain the damaged network part [4]. The presented approach in this paper is novel in the sense that both steps are combined within MoFoDatEv. Therefore, it is a powerful tool to evaluate data sets and also to localize leakages. In the case of the determination of the PDTP, all information on each pressure curve is densified to only one value. With MoFoDatEv, all information on each pressure curve remains for leakage localization as no densification to PDTP values is necessary. Therefore, MoFoDatEv can be used to determine the PDTP and to identify the affected EA. This is possible because knowledge of the leakage position is included and the time delays for all sensors are calculated with $\Delta_{i,L}$ during the quality assessment. In the case of an unknown leakage position, test leakages (TL) j can be placed over the whole DHN to determine the time delays $\Delta_{i,j}$. For each TL j, a $Q_{j}$ is determined and the TL j with the lowest $Q_{j}$ corresponds best to the model conception. This TL j is then chosen to be closest with respect to the leakage position. With this information, a ranking of the EAs can be generated if at least one TL j is placed in every EA. The network investigated here has a total trench length of around 90 km and 8250 pipes. In [4,12,21], TLs are placed in a 10 m grid with at least one TL per pipe. Therefore, 24,368 TLs j were placed throughout the network. In this work, calculation time is critical, as $Q_{j}$ has to be calculated for every TL j. The computing time was decreased as much as possible by using only one TL j in the middle of each EA in a supply pipe. Because the network is divided into 28 different EAs, there are 28 TLs placed in the whole network. Thus, MoFoDatEv has the advantage that both necessary steps, the determination of the PDTP and the attribution to EA, can be combined. This enables the use of all measurement data for localization directly and can lead to improvement of the quality of the results. MoFoDatEv is applied to real measurement data as well as artificial generated data.

## 5. Results and Discussion

The real events presented in Table 1 are evaluated with MoFoDatEv. The model is applied to each event separately. In Section 5.1, the quality of the data sets is assessed. To this end, a minimal $Q_{L}$ is searched for, as it shows how well the model fits the measurement data. For this purpose, the best combination of $T_{1}$ and $T_{2}$, selected from a given grid, is determined for each data set. For this combination, optimized values for $t_{1}$ and $\Delta_{p,t}$ are calculated. This evaluation is the basis for the classification of the datasets. For the classification of the data sets, further available parameters are included such as $\bar{\Delta_{p}}$, for example. In Section 5.2, MoFoDatEv is applied for leakage localization directly.

### 5.1. Quality Assessment of Several Real Events

The application of MoFoDatEv leads to optimal results for $t_{1}$ and $\Delta_{p,t}$ for each event. These parameters are optimized for the best time frame, based on $T_{1}$ and $T_{2}$, out of a given grid. To classify the different real events, Table 2 was created with the parameters $T_{1}$,$\;T_{2}$ for the optimal time frame selected from all combinations and $t_{1}$ for the start and $t_{2}$ for the end of the pressure drop. Furthermore, $\bar{\Delta_{p}}$ for the pressure level difference of the whole event and $\Delta_{p,t}$ for the time difference are presented.

**Figure 5:**
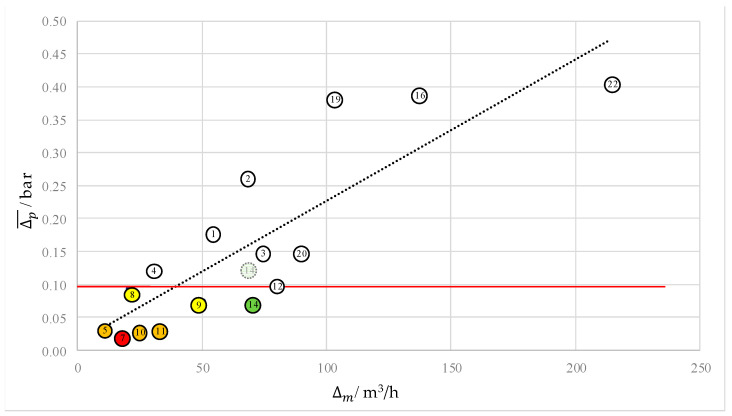
Comparison between pressure and refill mass flow level differences for each event.

Table 2 shows the events with the above-mentioned parameters. The table is sorted in ascending order with respect to $t_{1}$. In the first step, all events were neglected which have $t_{1}<-15\;s$ or $t_{1}>15\;s$. Larger absolute values mean that the pressure drop is extremely early before or after the LDTP and the difference between $t_{1}$ and the LDTP is too large considering the speed of sound and the network size. Therefore, the third criterion from Section 4.1 is not fulfilled and the PDTP and LDTP seem not to match in a physical sense. Furthermore, events with a negative value of $\bar{\Delta_{p}}$ are winnowed. Negative values lead to a pressure rise, not to a pressure drop. Based on all 22 events, only 16 events remain. For these 16 events, a comparison is performed between $\bar{\Delta_{p}}$ and $\Delta_{m}$. The results are shown in Figure 5.

The figure shows the pressure level difference $\bar{\Delta_{p}}$ found by MoFoDatEv with respect to the refill mass flow level difference $\Delta_{m}$ for each event. A strong correlation is visible, which is indicated by the black dashed line. The pressure level difference is linearly related to the refill mass flow level difference. Experience shows that leakage localization is very hard below a change in the refill mass flow of 50 $m^{3}/h$. It was assumed that pressure waves are not distinct enough in these cases. This matches well to the small pressure level differences extracted by MoFoDatEv in the case of these events. However, the greater the pressure level difference, the better the data set can be evaluated. Therefore, seven data sets (red, orange, yellow, and black) with $\bar{\Delta_{p}}<0.1\;\mathrm{bar}$ (red horizontal line) were considered in more detail. Figure 4 shows an example of sensor data within each of the seven data sets to get an impression of the pressure curves. As a result, the point for event 14 was shifted (light green dotted circle in Figure 5).

For the further analysis, these seven data sets are divided into four categories, which are presented in Table 3.

The table indicates how well the data sets correspond to MoFoDatEv. The color red means a low consistency with the model and a low evaluability whereas orange and yellow show middle and high consistency with the model, respectively, but also low evaluability. At the end, the color green leads to a middle consistency with the model and a high evaluability. For each of these seven data sets, the different pressure curves were evaluated in detail.

**Figure 6:**
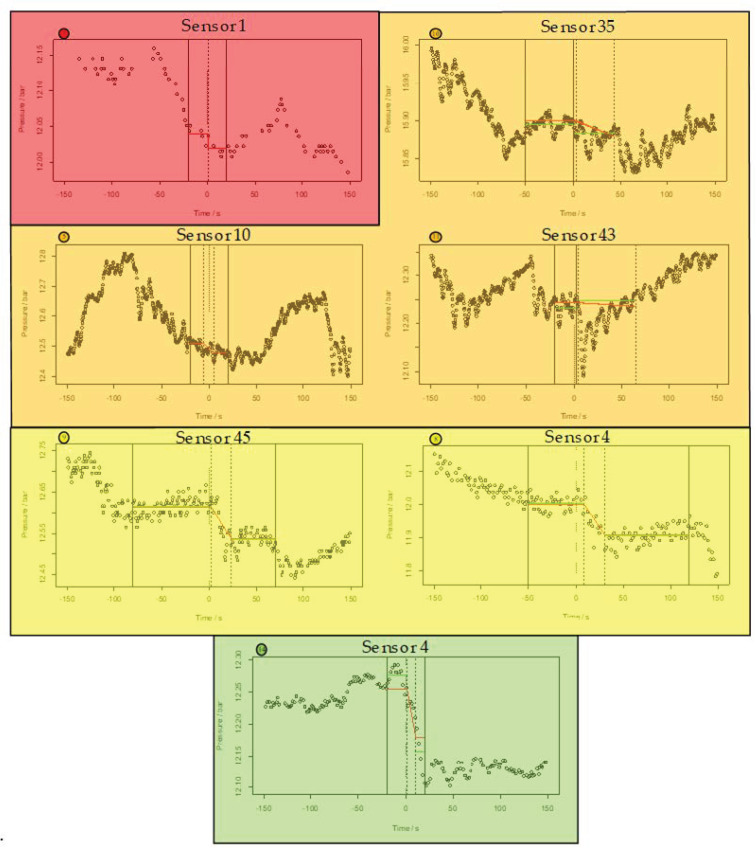
Pressure curves for one exemplary sensor for events where MoFoDatEv extracted small values of $\bar{\Delta_{p}}$.

Figure 6 shows the pressure curves for one selected sensor for each of the seven data sets where MoFoDatEv found a small pressure level difference $\bar{\Delta_{p}}$. MoFoDatEv can evaluate events with suitable results if the pressure level difference before and after the LDTP is large enough and if the change takes place reasonable fast. The seven events in Figure 4 are sorted from left to right and top to bottom in ascending order of their value of $\bar{\Delta_{p}}$. The pressure curves are evaluated to find the start of the pressure drop. To this end, evaluation is easier for pressure curves with larger $\bar{\Delta_{p}}$ values.

The first pressure curve is for sensor 1 from event 7 (top left, red). The data set does not match the model assumptions used in MoFoDatEv, hence the results of MoFoDatEv are not reliable. In contrast to $t_{1}=0.07$ and $\Delta_{p,t}=0.38$ found by MoFoDatEv, the pressure in fact declines in between −50 and +20 s, which is a rather long period of time. As mentioned previously, the PDTP and LDTP do not match in this case and the data set is not taken into account for further considerations. Altogether, this event is colored red (cp. Table 3) because the consistency with the model and the evaluability are low.

The next three events are number 10, 5, and 11, with selected sensors 35, 10, and 43 (top right and upper middle row, orange), respectively. Those three events present a middle consistency with the model but also a low evaluability. The refill process seems to be superposed by other processes in the DHN to a large extent. Furthermore, there are some large oscillations which lead to conspicuous pressure drops. Therefore, the pressure wave of the real event cannot be evaluated exactly. For event 11, the pressure values seem to be on the same level the whole time with only one exception around the LDTP. However, this is not interpretable because it does not correspond to the model conception with higher start than end values.

The following events, 9 and 8, with selected sensors 45 and 4 (lower middle row, yellow), respectively, show a high consistency with the model but a low evaluability. The pressure level difference is not very much larger than the noise of the pressure data. Furthermore, the data rate is low. Event 9 shows three plateaus and the pressure decreases slowly. A clear pressure drop is not visible which could be evaluated. Event 8 also shows no clear pressure drop. In the end, it seems that the pressure will further decrease.

The last dataset to be considered was event 14. The measurement data have a middle consistency with the model and a high evaluability. A clear pressure drop around the LDTP is recognizable. The pressure level difference found by MoFoDatEv is the largest here, with $\bar{\Delta_{p}}=0.076$. However, it is clearly visible that MoFoDatEv underestimates the real change in pressure by a factor of almost two.

Based on this analysis, for the further evaluation, the six events 5, 7, 8, 9, 10, and 11 are excluded. However, event 14 will be further considered. This event is also light green in Figure 6. Therefore, only 10 data sets are remaining. These 10 data sets are now compared with respect to the duration of the pressure drop $\Delta_{p,t}$ and the duration of the refill mass flow rise $\Delta_{m,t}$. In Figure 5, the results are presented.

**Figure 7:**
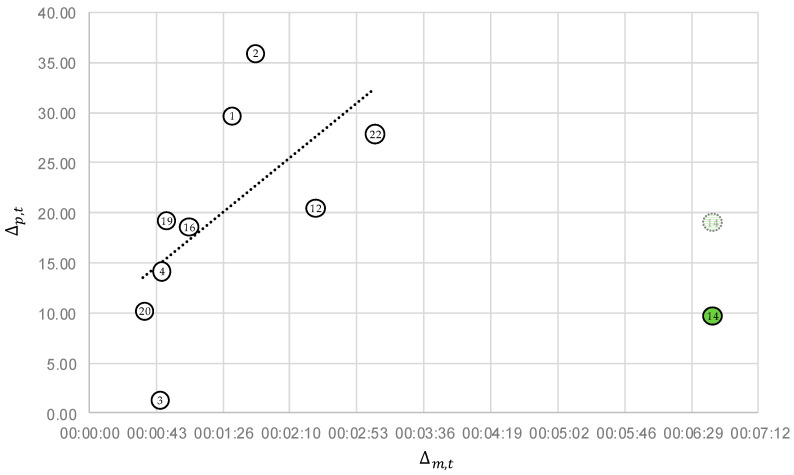
Comparison between the time for the pressure and the refill mass flow change.

Figure 7 shows the duration of the pressure drop $\Delta_{p,t}$ with respect to the duration of the refill mass flow rise $\Delta_{m,t}$. Both durations seem to show a correlation but take place on two totally different time scales. The duration of the refill mass flow rise is constituted in minutes and the duration of the pressure drop in seconds. For the duration of the pressure drop $\Delta_{p,t}$, the results were determined with MoFoDatEv. A strong correlation for the remaining ten data sets is visible. Large parts of the events are refill processes, which act like a real leakage on the entire network. The increase in the replenishment quantity during a refill process depends strongly on how the corresponding motor-driven or manually operated valve is moved. This can be opened quickly; hard and abruptly; or rather slowly, gently, and smoothly. In practice, both operations are possible because the management of leakages relies on the experience of the operators. The effects of a leakage are highly dependent on its position in the network [24]. In the case of a real leakage, a fast pressure change in a short time period is expected.

These differences can be seen exemplarily in events 20 and 22. In event 20, the duration of the refill mass flow rise (35 s) and the duration of the pressure drop (10 s) are very small. However, the replenished quantity is 100.5 $m^{3}/h$, which seems to be a larger leakage based on the refill mass flow (cp. Table 1). In contrast, for event 22, the duration of the refill mass flow rise is about 3 min and the duration of the pressure drop is 28 s, which is very long. The green event 14 (cp. Figure 6) is an outlier because MoFoDatEv extracts the values of $\bar{\Delta_{p}}$ and $\Delta_{p,t}$ inexactly. If better values are extracted manually, the point also shifts upward by a factor of two (light green). During this event, the duration of the refill mass flow rise is very long, at 6:41 min, whereas the duration of the pressure drop covers only a short time period of 10 s. This leads to the fact that the consistency with the model is middle but, in contrast, the evaluability is high, as shown in Table 3. Based on the detailed evaluation steps, all 22 events are classified in three different groups. The groups are presented in Table 4.

The table shows the 22 events divided into three different groups. Each group is sorted based on their values of $\bar{\Delta_{p}}$, in descending order. Additionally, the table contains the values for $\bar{\Delta_{p}}$, $g_{p}$, $Q_{L}$, $T_{1}$, $T_{2}$, and the length of the time window. The first group contains all events which have a high consistency with the model conception and also still have high evaluability. These were the remaining 10 best events. Each event has its own optimal time frame $T_{1}$,$T_{2}$ with different time lengths. The average length is 121 s and $T_{1}=-70\;s$ and $T_{2}=51\;s$ on average. Moreover, in the first group, all events have a high value of $\bar{\Delta_{p}}$, which indicates a high pressure difference between the start and end pressure values. Therefore, the pressure drop is expected to be better analyzable and these events can be well evaluated according to a small $Q_{L}$. The second group contains all events which have a worse consistency with the model and a low evaluability. In addition, the whole time frame starts later with $T_{1}=-40\;s$ but ends with $T_{2}=55\;s$ a bit later. These events have a low/middle/high consistency with the model conception and low evaluability (cp. Figure 6). This is also reflected by the smaller values of $\bar{\Delta_{p}}$. The third group contains all events which are neglected based on Table 2. For these events, the third quality criterion from Section 4.1 is not fulfilled and the PDTP and LDTP seem not to match in a physical sense. Furthermore, the events 6 and 21, with a negative value of $\bar{\Delta_{p}}$, are neglected. The value of $g_{p}$ is then also negative, which indicates a pressure rise instead of a pressure drop. This is not admissible. The evaluability with MoFoDatEv of these events is also not successful because the values of $Q_{L}$ are very high.

With the development of MoFoDatEv, it was possible to evaluate all 22 real events. Each event represents a leakage in the network. Most of them were refill processes, which act like a leakage on the network. Based on the different analysis steps, the events can be classified into three groups. Events which have a middle or high consistency with the model and also events which do not correspond to the model concept. MoFoDatEv cannot only be used to evaluate the events, but also to localize the leakages directly. The application is presented with artificial and real data in the following section.

### 5.2. Leakage Localization with MoFoDatEv

For the application of MoFoDatEv for leakage localization, the measurement data of event 22 are considered as well as artificial data. The overall best time frame is defined with $\left[T_{1},\;T_{2}\right]=\left[-60,\;60\right]$. For the generation of the artificial data, MoFoDatEv was applied to event 22. As a result, the values of $t_{1}$, $\Delta_{p,t}$, and $\bar{\Delta_{p}}$ are available. Furthermore, all $a_{i}$ and $e_{i}$ values are available. From the real data set, the time points and sensors are kept and only the pressure values are replaced with data generated by MoFoDatEv. These calculated values are then the “new real data”, the artificial data. For localizing the leakage, the aforementioned 28 TLs were placed in the whole network. For each TL *j*, the start value search is determined as described in Section 4.1. However, for the leakage localization, the overall best time frame is fixed and is not optimized. Therefore, only start values of $t_{1}$ and $\Delta_{p,t}$ will be searched over with the rough map and then optimized. For both data sets, artificial and measurement, the leakage localization is performed with perfect start values as well as a rough map for the start value search. The advantages are presented in Table 5.

**Figure 8:**
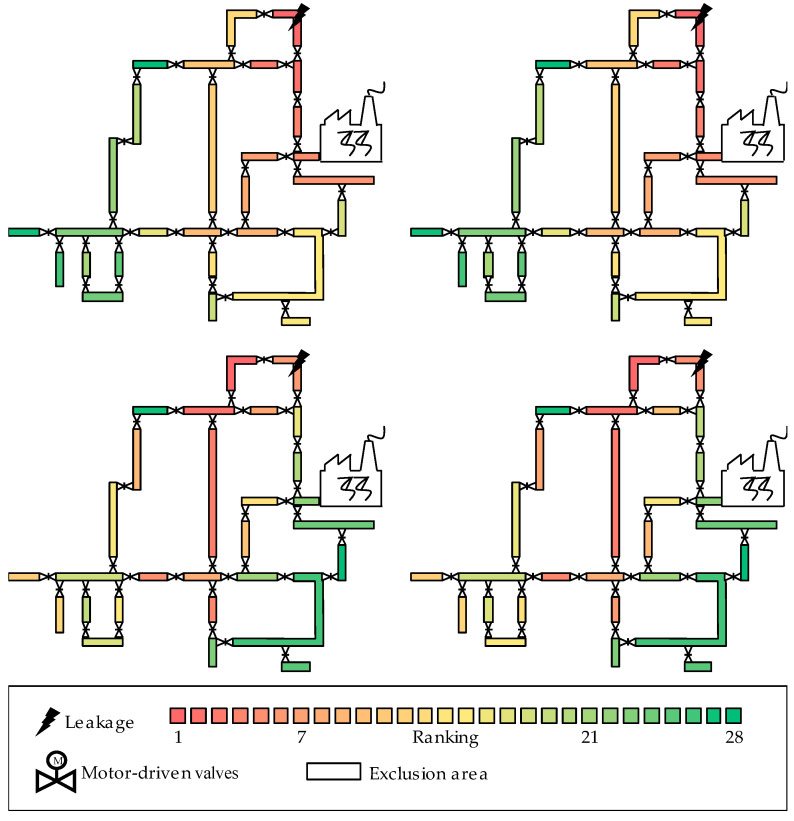
Result of leakage localization with MoFoDatEv with coloring of exclusion areas according to the ranking (**top left**: artificial data and perfect start values for optimization; **top right**: artificial data and search for start values for optimization; **bottom left**: measurement data and perfect start values for optimization; **bottom right**: measurement data and search of start values for optimization).

The table shows four levels of difficulty for the application of MoFoDatEv to localize leakages. The first case is the usage of artificial data with perfect start values to localize the leakage with MoFoDatEv. This is the easiest case and is used to check the model for the localization task. The perfect start values are exactly the same values which were used to generate the artificial data. If this execution is not possible with the artificial data, the measurement data would not be suitable either. The second case also uses artificial data, but the start values are searched for over the rough map. Then, these values are optimized and used for the localization. This is a little bit more difficult because it is not clear whether reasonable start values or the optimum can be found. More difficult is the third case, where perfect start values are used with the measurement data. If it is possible to evaluate the measurement data with perfect start values to achieve suitable results, then MoFoDatEv is robust and can work with artificial and real data. The last case, with a rough map for the start value search for the measurement data, is even more difficult. No preconditions or other information are available. The start values have to be determined directly from the measurement data over the rough map and following optimization. For all four cases, the leakage localization was performed. The results of the achieved ranking are presented in Figure 8 with a simplified network topology.

The figure shows the results of the leakage localization with MoFoDatEv with coloring of the EAs according to the ranking. For representation, the investigated DHN is shown with its simplified network topology and the right leakage of this event is located in the EA with the black lightning. The coloring indicates the ranking from Place 1 (red) to Place 28 (green) so that the leakage is in the respective EA. The color red means that this EA has the smallest $Q_{j}$ and the leakage will be in that EA. As the value for $Q_{j}$ increases, the color changes from red across orange and yellow to green. Top left presents the result of the artificial data with perfect start values for the following optimization. The EA affected by the leakage is found perfectly because it is colored red. The success of this case indicates that the model is now suitable for leakage localization and it could be continued to check the other cases. Top right shows the result of the artificial data with start value search and following optimization. For the data evaluation, the overall best time frame was determined (cp. Table 2). For this purpose, the values for of $t_{1}$ and $\Delta_{p,t}$ of the time frame $\left[T_{1},\;T_{2}\right]=\left[-60,\;60\right]$ were also calculated. These values are now the start values for the optimization, which leads also to the correct localization of the leakage in the EA containing the black lightning. Therefore, reasonable start values were found over the rough map. After the investigation with the artificial data, MoFoDatEv was also applied to the measurement data of event 22. Bottom left presents the result for the measurement data with the same perfect start values for the optimization as with the artificial data. The affected EA is located as the EA with the color red, which is not the correct one but it is also next to the right EA with the black lightning (color orange). The deviation could be explained with measurement noise within the real data, which has an influence on the correct evaluability. However, a suitable result is achieved. The last result is presented at the bottom right in the figure, which shows the result of the measurement data with a start value search and following optimization. Here, the affected EA by the leakage is also located by the EA with the color red, which is the wrong EA but is also next to the right EA with the black lightning (color orange).

With artificial data, it is proven that the idea fulfils the task for the leakage localization and the application of MoFoDatEv works. For practical purposes, an optimization with perfect start values is not possible because they are unknown. However, with a start value search over a rough map, the model can be applied to real data, which leads to much more plausible results in the neighborhood of the right EA for event 22. In fact, there is no difference working with perfect start values or with ones from a start value search. Due to these plausible results, MoFoDatEv was also applied to all other events based on Table 1. For each event, their start values were searched for with the rough map and then the optimization was performed. To compare all events, the aforementioned PC from Section 1 was used. The results are presented in Table 6.

The table shows the comparison of the leakage localization performance between the best BCP algorithm applied in [3] and MoFoDatEv. The BCP algorithm was applied in the optimal time frame of $\left[T_{1},\;T_{2}\right]=\left[-100,\;30\right]$, and for MoFoDatEv the time frame $\left[T_{1},\;T_{2}\right]=\left[-60,\;60\right]$ was selected. All 22 events were also categorized similarly to the result of Table 4. Based on the PC values, all events were evaluated differently. The range of the PC is, for the BCP algorithm, from 100.0% (first place in the ranking) to 35.7% and, for MoFoDatEv, from 100.0% to 3.6% (last place in the ranking). With an average value of 77.3% for the BCP algorithm and 74.4% for MoFoDatEv over all events, suitable leakage localization results were achieved. Therefore, this novel method is not much worse than the BCP algorithm from the literature. In five cases, the affected EA by the leakage is in the first place. In contrast, BCP ranks the affected EA in the first place only for four events. Furthermore, for ten events, the right EA is in the first three places in the ranking (PC ≥ 92.9%) with MoFoDatEv. Only nine events are in the first places in the ranking with the BCP algorithm. However, events 9 and 10 show that there could be some improvements necessary within MoFoDatEv to reach higher PC values. The three different groups are established based on the quality assessment in Section 5.1. If only the best ten events within the first group were considered, then MoFoDatEv achieved a higher average PC of 79.3% compared to 72.9% for the BCP algorithm. Only three times, for events 14, 20 and 22, did BCP reach a higher PC value. Within the second group, the BCP algorithm is, with 86.3%, on average better than MoFoDatEv, with 62.5%. For events 5, 9, and 11, the affected EA were ranked in the first place with the BCP algorithm. These three events were neglected, based on Figure 6, for the following evaluation with MoFoDatEv, because the pressure difference $\bar{\Delta_{p}}$ was not high enough. Therefore, events 9 and 10 reached only a PC value of 7.1% and 3.6%, respectively. The last group is based on the analysis of Table 1. The value of $t_{1}$ was inadmissible and the value of $\bar{\Delta_{p}}$ was negative. BCP reaches 75.6% on average in this group whereas MoFoDatEv reaches a better value of 81.5%. This illustrates that, theoretically, even these data sets can be evaluated and lead to a suitable ranking result. It could be possible that the LDTP was determined falsely. If the pressure difference is high enough, then MoFoDatEv should be used for leakage localization because more information remains and both evaluation steps for the determination of the PDTPs and the attribution to EA could be combined.

## 6. Conclusions

In the case of a large spontaneous leakage, fast separation of the damaged network part is necessary. Hence the pressure wave can be evaluated to decide which EA to separate. Results of the leakage localization are of different quality when evaluating 22 recorded events. For the evaluation of several real events, MoFoDatEv was developed and applied. MoFoDatEv contains prior knowledge and a simplified physical model for evaluating the pressure wave in the case of large spontaneous leakages. MoFoDatEv requires the four parameters $T_{1},T_{2}$, $t_{1}$, and $\Delta_{p,t}$ to be optimized (note that $\bar{\Delta_{p}}$ cannot be optimized separately as this parameter is dependent on the time intervals). If the model fits the real data with the smallest deviation possible, the best suitable values are found.

For quality assessment, three quality criterions could be used to rate the quality of the data sets after the 22 real events were fitted with MoFoDatEv. In the first step, six events with inappropriate values for the value of $t_{1}$ were neglected. The values are supposed to be in the range of $-15<t_{1}<15$ for physical reasons. In the second step, the values of the pressure $\bar{\Delta_{p}}$ and the refill mass flow level difference $\Delta_{m}$ of 16 events were considered. A linear correlation between the pressure level difference and the refill mass flow level difference was found. Below a refill mass flow difference of 50 $m^{3}/h$ and a pressure level difference of $\bar{\Delta_{p}}<0.1\;\mathrm{bar}$, seven events were considered in detail, as practical experience indicates bad evaluability in these cases. Consistency with the model was checked and a further six events were neglected. Thus, a refill mass flow difference of 50 $m^{3}/h$ is set as the lower limit for evaluability for the network and data considered here. A second linear correlation between the duration of the changes of pressures and the refill mass flow is indicated by the data. The remaining ten events were categorized into three different groups based on the results of the quality assessment.

For the localization of leakages, a ranking for possible leakage localizations was determined with MoFoDatEv accordingly. The results were obtained for artificial and measurement data. MoFoDatEv is compared to the BCP algorithm from [3] for the application on the measurement data. It has to be taken into account that the best overall timeframe is not yet determined for MoFoDatEv. Over all events, the BCP leads, on average, to a higher PC value of 77.3%, compared to 74.4% for MoFoDatEv. However, the model assesses the quality of the events and, in the first group with the remaining ten best events, MoFoDatEv reached a PC of 79.3%, compared to 72.9% with the BCP algorithm. Therefore, if a pressure drop is available and the value of $\bar{\Delta_{p}}$ is large enough, MoFoDatEv leads to better results because all information is considered and there is no densification where information could be lost.

Future work will focus on an improvement of MoFoDatEv. The leakage localization especially has a great potential to reach even better results for all events. As in [3], a best overall time frame should be determined. It is perhaps possible to fit not the whole piecewise function but only the linear decrease for the pressure drop. Then, only the start point and the duration of the pressure drop will be determined and fitted to the measurement data. With this improvements, higher PC values could be possible.

## Figures and Tables

**Table 1:** Evaluation of 22 real events (Adapted with permission from Ref. [3]. 2022, Vahldiek).

#	Date	Begin	$\mathrm{Quantity}\;\left[m^{3}/h\right]$	End	$\mathrm{Quantity}\;\left[m^{3}/h\right]$	$\Delta_{m}\;\left[m^{3}/h\right]$	$g_{m}\;\left[m^{3}/h/min\right]$	$\mathrm{PC}\;\mathrm{BCP}\;[T_{1}=-100,\;T_{2}=30]$
1	27.07.2017	17:38:00	23.5	17:39:30	78.0	54.4	36.3	35.7%
2	27.07.2017	17:42:45	78.0	17:44:30	146.1	68.2	39.0	46.4%
3	27.07.2017	17:51:30	146.1	17:52:15	221.4	75.2	100.3	85.7%
4	27.07.2017	17:54:30	221.4	17:55:15	251.9	30.6	40.8	57.1%
5	01.08.2017	08:23:24	84.0	08:24:00	94.0	10.0	16.7	100.0%
6	01.08.2017	09:02:00	46.1	09:09:30	113.5	67.4	9.0	78.6%
7	01.08.2017	13:49:26	83.0	13:49:55	100.0	17.0	35.2	60.7%
8	26.06.2018	13:31:46	29.0	13:32:35	50.0	21.0	25.7	82.1%
9	26.06.2018	13:33:54	50.0	13:35:45	100.0	50.0	27.0	100.0%
10	26.06.2018	13:36:34	100.0	13:36:55	124.0	24.0	68.6	75.0%
11	26.06.2018	13:37:17	124.0	13:37:53	158.0	34.0	56.7	100.0%
12	25.07.2018	06:53:26	60.0	06:55:52	140.0	80.0	32.9	92.9%
13	09.05.2020	13:14:11	70.8	13:15:13	231.7	160.8	155.6	100.0%
14	03.09.2020	14:34:49	6.1	14:41:30	76.9	70.9	10.6	92.9%
15	17.09.2020	17:46:19	27.1	17:48:53	195.6	168.6	65.7	85.7%
16	25.09.2020	10:58:13	21.5	10:59:20	158.4	136.9	122.6	35.7%
17	29.09.2020	09:45:23	9.4	09:45:30	421.3	411.9	3530.0	85.7%
18	28.07.2021	16:59:25	43.8	16:59:53	122.0	78.1	167.4	53.6%
19	28.07.2021	17:02:40	46.9	17:03:30	149.9	103.0	123.6	92.9%
20	28.07.2021	17:30:46	100.5	17:31:21	200.7	90.0	154.3	92.9%
21	30.09.2021	09:28:47	90.0	09:28:58	133.0	43.0	234.5	50.0%
22	07.10.2021	08:14:08	39.8	08:17:12	254.2	214.4	69.9	96.4%

**Table 2:** Real events sorted by $t_{1}$.

#	Date	$T_{1}$	$t_{1}$	$\Delta_{p,t}$	$T_{2}$	$\bar{\Delta_{p}}$
18	28.07.2021	−100	−60.92	39.95	20	0.0736
13	09.05.2020	−120	−47.88	71.07	40	0.5341
17	29.09.2020	−90	−30.61	10.10	20	0.0050
15	17.09.2020	−50	−29.33	52.62	70	0.3178
2	27.07.2017	−120	−10.27	36.06	90	0.2652
6	01.08.2017	−30	−10.01	10.28	40	−0.0054
5	01.08.2017	−20	−9.91	11.32	20	0.0325
12	25.07.2018	−30	−8.98	20.44	20	0.1023
1	27.07.2017	−120	−8.52	29.85	110	0.1837
22	07.10.2021	−110	−7.88	27.88	50	0.4028
19	28.07.2021	−50	−7.87	19.67	20	0.3836
16	25.09.2020	−20	−4.48	18.68	20	0.3905
4	27.07.2017	−20	−2.92	14.31	20	0.1242
3	27.07.2017	−120	−2.13	1.39	120	0.1536
14	03.09.2020	−20	−0.85	9.72	20	0.0764
10	26.06.2018	−50	−0.25	0.30	40	0.0278
20	28.07.2021	−90	−0.19	10.27	40	0.1517
7	01.08.2017	−20	0.07	0.31	20	0.0211
9	26.06.2018	−80	0.08	20.22	70	0.0733
11	26.06.2018	−20	1.79	0.31	60	0.0335
8	26.06.2018	−50	5.52	21.68	120	0.0925
21	30.09.2021	−20	9.11	0.35	80	−0.1510

**Table 3:** Categories for data sets with a small pressure level difference.

Color	Consistency with Model	Evaluability
Red	Low	Low
Orange	Middle	Low
Yellow	High	Low
Green	Middle	High

**Table 4:** Three different groups to classify all 22 events.

#	$\bar{\Delta_{p}}$	$g_{p}$	$Q_{L}$	$T_{1}$	$T_{2}$	Length
22	0.4028	0.0145	0.0044	−110	50	160
16	0.3905	0.0209	0.0064	−20	20	40
19	0.3836	0.0195	0.0071	−50	20	70
2	0.2652	0.0074	0.0019	−120	90	210
1	0.1837	0.0062	0.0008	−120	110	230
3	0.1536	0.1105	0.0072	−120	120	240
20	0.1517	0.0148	0.0025	−90	40	130
4	0.1242	0.0087	0.0012	−20	20	40
12	0.1023	0.0050	0.0003	−30	20	50
14	0.0764	0.0079	0.0008	−20	20	40
Mean				−70	51	121
8	0.0925	0.0042	0.0009	−50	120	170
9	0.0733	0.0036	0.0008	−80	70	150
11	0.0335	0.1081	0.0012	−20	60	80
5	0.0325	0.0029	0.0028	−20	20	40
10	0.0278	0.0926	0.0008	−50	40	90
7	0.0211	0.0680	0.0003	−20	20	40
Mean				−40	55	95
13	0.5341	0.0075	0.0073	−120	40	160
15	0.3178	0.0060	0.0051	−50	70	120
18	0.0736	0.0018	0.0019	−100	20	120
17	0.0050	0.0005	0.0023	−90	20	110
6	−0.0054	−0.0005	0.0025	−30	40	70
21	−0.1510	−0.4315	0.0138	−20	80	100
Mean				−68	45	113

**Table 5:** Comparison for usage of the perfect start values and the start value search for the artificial and measurement data.

	Perfect Start Values	Rough Map for Start Value Search
Artificial data	I: Simplest case as a prerequisite for application to measurement data.	II: Possibility to find suitable start values.
Measurement data	III: Possibility to evaluate measurement data with perfect start values.	IV: No preconditions are used and start values have to be determined from measurement data.

**Table 6:** Comparison of the PC results for leakage localization with a BCP algorithm and MoFoDatEv.

#	$\mathrm{PC}\;\mathrm{BCP}\;\left[T_{1},\;T_{2}\right]=\left[-100,\;30\right]$	$\mathrm{PC}\;\mathrm{MoFoDatEv}\;\left[T_{1},\;T_{2}\right]=\left[-60,\;60\right]$
22	96.4%	85.7%
16	35.7%	89.3%
19	92.9%	100.0%
2	46.4%	92.9%
1	35.7%	92.9%
3	85.7%	92.9%
20	92.9%	17.9%
4	57.1%	96.4%
12	92.9%	100.0%
14	92.9%	25.0%
**Mean**	**72.9%**	**79.3%**
8	82.1%	96.4%
9	100.0%	7.1%
11	100.0%	89.3%
5	100.0%	100.0%
10	75.0%	3.6%
7	60.7%	78.6%
**Mean**	**86.3%**	**62.5%**
13	100.0%	100.0%
15	85.7%	85.7%
18	53.6%	100.0%
17	85.7%	78.6%
6	78.6%	89.3%
21	50.0%	35.7%
**Mean**	**75.6%**	**81.5%**
Mean overall	77.3%	74.4%

## Data Availability

Not applicable.

## Abbreviations

Symbol	Description
**Glossary**	
BCP	Bayesian single change point algorithm
DHN	District Heating Network
EA	Exclusion Area—specific area in the network which can be separated
LDTP	Leakage detection time point
PC	Performance criterion—comparison metric
PDTP	Pressure drop time point
QM	Quality measure
TL(s)	Test leakage(s)—position(s) used for leakage localization
**Nomenclature**	
i	Specific sensor
N	Number of evaluated sensors per event
L	Leakage
$p_{i}\left(t\right)$	Non-uniform time-dependent pressure value of a specific sensor i
$q_{i}\left(t\right)$	Fitted non-uniform time-dependent pressure value of a specific sensor i
$\left[T_{1},T_{2}\right]$	Evaluation time frame with a left and right time border
$t_{1}$	Start of the pressure drop at the sensor closest to the leakage position
$t_{2}$	End of the pressure drop at the sensor closest to the leakage position
$t_{1,i}$	Start of the time-shifted pressure drop at a specific sensor i
$t_{2,i}$	End of the time-shifted pressure drop at a specific sensor i
$\Delta_{i,L}$	Time delay of a specific sensor i with respect to the sensor closest to the leakage position L
$a_{i}$	Mean value for the start values of a specific sensor i
$e_{i}$	Mean value for the end values of a specific sensor i
$\Delta_{p,i}$	Pressure level difference of a specific sensor i
$\Delta_{d,i}$	Difference between MoFoDatEv and the measurement data of a specific sensor i
$\bar{\Delta_{p}}$	Pressure level difference of the pressure drop
$\Delta_{p,t}$	Duration of the pressure drop
$g_{p}$	Gradient of the pressure drop
$\Delta_{m}$	Refill mass flow level difference of the refill mass flow rise
$\Delta_{m,t}$	Duration of the refill mass flow rise
$dp_{i}$	Evaluated data points of a specific sensor i in the evaluation time frame
$dp_{all}$	Evaluated data points for all sensors of the whole data set in the evaluation time frame
$Q_{L,i}$	Quality measure of the fit for a specific sensor i and MoFoDatEv for the leakage position L
$Q_{L}$	Quality measure of the fit for all sensors of the whole data set and MoFoDatEv for the leakage position L
j	Number of the test leakage
$\Delta_{i,j}$	Time delay of a specific sensor i with respect to the sensor closest to a test leakage j
$Q_{j}$	Quality measure of the fit for all sensors of the whole dataset and MoFoDatEv for a test leakage j
